# Explaining the Self‐Care Behaviors in Type 2 Diabetic Patients Based on Adverse Childhood Experiences, Subjective Socioeconomic Status, and Mindfulness in Isfahan, Iran

**DOI:** 10.1002/hsr2.70952

**Published:** 2025-06-20

**Authors:** Mehdi Nosratabadi

**Affiliations:** ^1^ Health and Social Welfare, Social Determinants of Health Research Center Isfahan University of Medical Sciences Isfahan Iran

**Keywords:** adverse childhood experiences, self‐care behaviors, subjective socioeconomic status, Type 2 diabetic

## Abstract

**Background and Aims:**

As reported by the World Health Organization, the prevalence of Type 2 diabetes has increased fourfold since 1980, with approximately 422 million individuals affected globally. Projections indicate that the number of people living with Type 2 diabetes could more than double in the next two decades. Given the expected increase in the prevalence of Type 2 diabetes in Iran, it is crucial to conduct a thorough investigation to evaluate the primary psychosocial factors associated with individuals suffering from Type 2 diabetes. Therefore, this study aims to investigate the relationship between mindfulness, subjective socioeconomic status (SES), and adverse childhood experiences (ACEs) in the context of self‐care practices among Type 2 diabetes patients in Isfahan, Iran.

**Methods:**

A cross‐sectional study involving 350 Type 2 diabetic patients, aged 20–44, was undertaken in 2023. Participants were randomly selected from six comprehensive health centers located in Isfahan. Summary of Diabetes Self‐Care Activities (SDSCA) measure, Brown's Mindful Attention Awareness Scale, ACEs module (CDC, 2010), and socio‐demographic factors, including age, gender, education, and subjective SES, were employed to collect data. The data was analyzed using a multivariate logistic regression model.

**Results:**

The study results indicate that 58.6% of all participants reported experiencing at least one ACE, while 50.9% of individuals with Type 2 diabetes exhibited inadequate self‐care practices. After adjusting for demographic factors, individuals who reported experiencing one to four ACEs (OR: 0.38; 95% CI: 0.17–0.63) and those with six or more ACEs (OR: 0.32; 95% CI: 0.15–0.78) exhibited a higher likelihood of engaging in poor diabetes self‐care behaviors compared to those who had no ACEs.

**Conclusions:**

Research suggests a strong correlation between childhood adversity and suboptimal self‐care practices in Type 2 diabetic patients. This finding underscores the importance of collaborative efforts between healthcare and social service organizations to implement effective screening, prevention, and early intervention strategies for individuals with Type 2 diabetes who have experienced ACEs.

**Abbreviations**
ACEs: adverse childhood experiences
SES: socioeconomic status
SPSS: statistical package for social sciences

## 1 Introduction

Type 2 diabetes represents around 90% of the overall diabetes burden, and the ongoing rise in diabetes prevalence globally can primarily be attributed to the growth of Type 2 diabetes and its related risk factors, including overweight and obesity, lack of physical activity, and high caloric consumption. This trend is further exacerbated by unhealthy dietary patterns linked to urbanization and a more sedentary way of life [1]. Current projections indicate that by the year 2045, the global population of individuals diagnosed with Type 2 diabetes is expected to exceed 700 million [2].

Considering the anticipated rise in the incidence of this condition and its implications on various demographic groups, Type 2 diabetes has emerged as one of the most intricate health challenges of the 21st century [3].

The incidence of Type 2 diabetes mellitus in Iran has shown a persistent rise in recent years, attributed to factors such as population growth, an aging demographic, urban development, increased rates of obesity, and a more sedentary lifestyle [4]. A recent study conducted in Iran has shown that men were predicted to have a Type 2 diabetes prevalence of 10.80%, while women were assessed to have a prevalence of 13.4% [5].

While individuals with Type 2 Diabetes are capable of effectively managing their self‐care routines, psychosocial factors have been identified as crucial elements that can improve diabetic self‐care practices [6]. This aspect warrants further investigation among Type 2 diabetic patients in Iran. Successful Type 2 diabetes management necessitates ongoing and active participation from patients. Most complications and issues related to managing diabetes arise from noncompliance with self‐management guidelines [7]. Type 2 diabetes is a long‐term condition that necessitates ongoing self‐management throughout an individual's life. Successfully managing Type 2 diabetes involves the adoption of intricate self‐care practices, which include making lifestyle modifications, adhering to dietary guidelines, engaging in regular physical activity, utilizing prescribed medications, monitoring blood glucose levels, and reducing smoking habits [8].

Studies demonstrate a strong link between traumatic experiences in childhood and the subsequent onset of Type 2 diabetes in adulthood [9, 10]. Adverse childhood experiences (ACEs) encompass not only detrimental home situations, like residing with a parent who struggles with substance abuse, a mentally ill individual, growing up in a single‐parent household or without parents, or being surrounded by criminal activity but also negative actions aimed at the child, including emotional, physical, and sexual abuse [11].

Studies indicate that experiencing adverse childhood events significantly contributes to a decline in health during adulthood [12, 13]. As stated in the basic theoretical Model of ACEs [14], there is a correlation between negative experiences and detrimental health consequences, including illness, which can be attributed to emotional dysregulation and ineffective coping strategies linked to stressful situations. These factors may lead to enduring distress [15]. The activation of the hypothalamic‐pituitary‐adrenal axis can significantly impact emotional, cognitive, and social development, thereby contributing to an increased susceptibility to diseases in later stages of life [16].

Mindfulness is characterized as a deliberate state of focused awareness where individuals accept their current experiences without judgment, remaining fully cognizant of the present moment [17]. Mindfulness practices demonstrate beneficial therapeutic effects and contribute to lasting outcomes for various chronic illnesses, including diabetes [18]. Indeed, individuals who practice mindful living and possess mindfulness skills, along with incorporating mindfulness‐based principles such as acceptance, are more inclined to engage in self‐cultivating behaviors. [19, 20]. Besides, for those who have experienced ACEs, mindfulness‐based interventions (MBIs) can enhance the application of coping strategies and boost overall mental well‐being by helping individuals identify and address the negative thoughts and feelings that often arise from such experiences [21]. Studies have shown that targeted MBIs can effectively help adult survivors of ACEs learn to acknowledge and explore their thoughts and emotions associated with past hardships [22, 23]. However, there is limited evidence regarding this relationship in the context of chronic diseases like Type 2 diabetes.

Subjective socioeconomic status (SES), defined as an individual's perception of their social standing and economic and financial influence, is associated with health outcomes beyond the objective indicators of SES [24]. Subjective SES encapsulates individuals' self‐assessments regarding their standing within the social hierarchy, thereby functioning as a psychological mechanism. [25]. An increasing number of studies indicate that individuals who perceive themselves as inferior to others are at a heightened risk for health issues compared to those who do not harbor such feelings of inferiority. These individuals tend to exhibit lower self‐esteem, experience higher levels of stress [26], and demonstrate suboptimal self‐management behaviors related to Type 2 diabetes, including a reduced likelihood of implementing a rigorous insulin regimen [27].

To the best of our knowledge, there is a lack of studies exploring the impact of psychosocial factors on self‐care practices among individuals with Type 2 diabetes in Iran. Consequently, this study aims to investigate the hypothesis that self‐care behaviors in Iranian patients with Type 2 diabetes are associated with ACEs, subjective SES, and mindfulness as key psychosocial factors.

## 2 Methods

### 2.1 Study Design and Sampling

This cross‐sectional survey research was carried out in Isfahan, Iran. The survey consisted of face‐to‐face administration of the questionnaire by two interviewers (an in‐person or self‐completed questionnaire). A total of 350 diabetic patients were randomly selected using Cochran's formula with a standard error of 0.05 (*d* = 0.05) and a Type I error of 0.02 (*α* = 0.02) and an average prevalence of Type 2 diabetic in Iran (*p* = 20%).

The target population for this study consisted of all diabetic patients who were referred to the comprehensive health centers affiliated with Isfahan Health Centers No. 1 and 2 (a total of 50,837 patients with Type 2 diabetes). The study employed a multi‐stage sampling method. Initially, all Isfahan Health Centers were categorized into two groups: Group 1 (comprising 30 comprehensive health centers with 28,480 Type 2 diabetic patients) and Group 2 (comprising 36 comprehensive health centers with 22,357 Type 2 diabetic patients). Subsequently, three comprehensive health centers were randomly selected from each group. Finally, a list of diabetic patients from each of the six chosen centers was compiled for inclusion in the study. The study participants, representing a diverse diabetic population, were selected through a randomized process utilizing a number table. This method ensured that the sample accurately reflected the demographic characteristics of the overall patient population, particularly in terms of gender and age.

A simple random sample was generated using a random number table. This process involved assigning unique numbers to each member of the population, ranging from 1 to N. The total population size and desired sample size were then determined. A starting point was selected within the random number table, and the sequence of reading numbers proceeded from left to right. Numbers from the table with the last three digits ranging from 0 to 350 were utilized, with an initial value of n set to 3. This process continued until the desired sample size was reached. Data collection took place at six different centers, with an average of 58 questionnaires completed at each center, resulting in a total of 350 patient responses. The data was gathered between November 2022 and March 2023.

To participate in this study, individuals must fulfill the following inclusion criteria: *A confirmed diagnosis of Type 2 diabetes. *Age 18 years or older. *Demonstrate satisfactory mental and psychological health. *Possess a minimum of 5 years of experience managing Type 2 diabetes. *Have no documented history of macrovascular complications, such as cardiovascular disease, stroke, or peripheral vascular disease.

Participants with physical disabilities, Type 1 or gestational diabetes, or those who declined to provide informed consent were excluded from the study. Informed consent was obtained through a process that ensured the sharing of information and addressed the questions of patients. Additionally, the voluntary nature of the decision regarding participation in the research was emphasized.

The research obtained the necessary ethical approval from the Research Ethics Committee of Isfahan University of Medical Sciences in Isfahan, Iran, under Ethics Code No. IR.MUI.REC.1399.373. Additionally, participants provided written informed consent, which guarantees that their identities and responses will remain anonymous and that their data will be handled with the utmost confidentiality.

### 2.2 Measures

In this study, the primary outcome variable was self‐care behaviors. The predictor variables included ACEs, subjective SES, and mindfulness. Additionally, gender, marital status, the education of women, and the education of husbands were treated as confounding variables.

### 2.3 Self‐Care Behavior

The Summary of Diabetes Self‐Care Activities (SDSCA) is a comprehensive instrument designed to evaluate diabetes self‐management practices. The SDSCA questionnaire was created by Toobert et al. [28]. The SDSCA evaluates six components related to the frequency of adherence to various self‐care practices, including exercise (2 questions), dietary choices (5 questions), blood glucose monitoring (2 questions), foot care (4 questions), Insulin injections or oral antidiabetic medications (1 question) and smoking habits (1 question), over the past week.

The SDSCA questionnaire consists of 15 items, each assessed on a Likert scale from 0 to 7. A lower score indicates less effective self‐care practices. The total score is derived by aggregating the individual scores from all questions, yielding a cumulative score that spans from 0 to 99. The internal consistency coefficient of the instrument is 0.82 in the English version [29] and 0.82 in Iranian studies as well [30].

### 2.4 Subjective SES

The baseline questionnaire incorporates the MacArthur Scale of Subjective Social Status [31]. This validated tool evaluates individuals' perceptions of their SES in relation to others, taking into account variables such as financial assets, educational levels, and job positions. Participants were shown a visual representation of a ladder with 10 steps and were asked to identify their relative position on the ladder compared to others. Each step is designated a numerical score ranging from 1 to 10. A mark placed on the lowest step is given a score of 1, while a mark on the middle step is awarded a score of 5. Conversely, a mark on the highest step corresponds to a score of 10. In the current study, for analysis, the categories were grouped in pairs: “*very bad and bad*” (1–3), “*regular*” (4–6), “*good and very good*” (7 or more). This scale has good psychometric properties in the Iranian population (test‐retest reliability = 0.78, Spearman's Rank‐Order Correlation Coefficient between Income and SSS = 0.74) [32].

### 2.5 ACEs Module

This study used the ACEs module as implemented in the Behavioral Risk Factor Surveillance System (BRFSS) to measure ACEs [33]. A checklist consisting of 11 items was employed to evaluate ACEs. Participants were asked whether these negative events transpired during their childhood, specifically before reaching the age of 18. The experiences examined included residing with a family member suffering from mental illness, incidents of physical abuse, sexual abuse, emotional abuse, domestic violence, parental separation or divorce, and the incarceration of a family member. The study's measurement focused not on the quantity of adverse events experienced, but rather on the participants' perception of a particular type of adverse event. Responses were collected using a scale with options including “never,” “once,” “more than once,” “don't know,” and “have no opinion.” Participants who responded “at least once” or “more than once” were categorized as “Yes,” while those who responded “never” were categorized as “No.” Individuals who selected “don't know” or “have no opinion” (*n* = 15) were excluded from further analysis [34, 35]. The classification of ACEs is informed by the prevalence and diversity of such events, as well as existing research on the various methodologies for categorizing ACEs [36, 37]. The counts of ACEs were classified as: no ACEs, one to five ACEs, and six or more ACEs [38].

As a result, the number of adverse childhood events was rated to be zero, one to five, or six or more. All abuse items (physical, emotional, and sexual), family dysfunction, and the overall scale score have kappa values in “BRFSS” ACEs scale ranging from 0.41 to 0.86, indicating that the test‐retest reliability appears to be adequate [39, 40].

### 2.6 Mindfulness Questionnaire

This brief questionnaire gauges the respondent's current mental state. The five‐question assessment explores their level of awareness, focus, and attention, emphasizing acceptance and non‐judgmental observation of the present moment. This tool has five questions, scaled from 0 (*not at all*) to 6 (*very much*). The scoring methodology for this tool requires that all items be reverse‐scored to accurately reflect a higher level of mindfulness. Subsequently, the average of the five values must be calculated to determine the overall score. The questionnaire, along with its simplified version, has demonstrated robust psychometric properties [41]. This tool has desirable psychometric properties in Iranian studies [42].

### 2.7 Demographic Variables

The demographic variables considered in this study included age, categorized into three groups: 18–22 years, 23–27 years, and 28–34 years. Additionally, gender was recorded as either female or male. Marital status was also examined, with categories including single, married, widowed, and divorced. Furthermore, the educational attainment of both mothers and fathers was evaluated, scored on a scale of 1–3, which encompassed illiterate, high school or diploma, and university education levels.

### 2.8 Data Analysis

This study employed descriptive analyses to examine the distribution of participant characteristics. Frequencies and percentages were calculated for ordinal variables, including education level, subjective SES, and the number of ACEs. Similarly, frequencies and percentages were determined for nominal variables such as gender, marital status. Continuous variables, such as duration of diabetes and mindfulness, were summarized using means and standard deviations. Bivariate analysis with the A Chi‐square test was utilized to assess the significant differences between ACEs and self‐care behaviors in diabetic patients, with a significance threshold set at 0.05. To ascertain the genuine factors influencing self‐care behaviors, the initial step involved identifying significant variables through the Chi‐square test. These variables were subsequently incorporated into a multivariate logistic regression model employing the forward selection method. The threshold for the univariate binary logistic regression analysis was established at ≤ 0.2. Analyses were adjusted for potential confounders, including gender, marital status, education of woman, and education of the husband. All analyses were completed using SPSS version 21.

## 3 Results

### 3.1 Sample Characteristic Descriptive

The analysis of the research data reveals that a significant portion of diabetic patients falls within the 35–44 age group, accounting for 58.3%. Additionally, the majority of these patients are female, comprising 65.4%, and most are married, representing 65.7% of the population studied.

A significant proportion of diabetic patients, specifically 50.9%, exhibited poor self‐care behaviors. Additionally, a considerable number of diabetic patients, representing 32%, reported experiencing five or more ACEs. Table 1 provides a comprehensive overview of the demographic characteristics of the diabetic patient population.

**Table 1:** Description of sample demographic characteristics (mean [SD]) or % by bender (*N* = 350).

Variables	% or Mean ± standard deviation
Age categories	
20–27 years old	63 (17.7%)
28–34 years old	84 (24%)
35–44 years old	204 (58.3%)
Gender	
Female	229 (65.4%)
Male	121 (34.6%)
Education of male patients	
Illiterate	89 (25.4%)
High School and Diploma	177 (50.6%)
University education	82 (23.4%)
Education of female patients	
Illiterate	100 (28.6%)
High School and Diploma	129 (36.9%)
University education	84 (24%)
Marital status	
Married	230 (65.7%)
Single	56 (16%)
Widowed	46 (13.1%)
Divorced	18 (5.1%)
Duration of diabetes	
Male patients	6.30 ± 4.36
Female patients	4.89 ± 3.23
Subjective socioeconomic status score	
1–3	138 (39.4%)
4–6	140 (40%)
7 or more	72 (20.6%)
Diabetes self‐care behavior	
Above the cut‐off*	169 (48.3%)
Below the cut‐off	178 (50.9%)
Number of ACEs	
No ACE	33 (9.4%)
One to four ACE	205 (58.6%)
Five or more ACE	112 (32%)
Mindfulness	
Score of male patients	15.14 ± 6.84
Score of female patients	13.12 ± 6.83

* Median = 24.

### 3.2 Bivariate Analyzes

Diabetic individuals with a greater prevalence of ACEs exhibited diminished engagement in diabetes self‐management practices, as evidenced by scores falling below the established threshold. According to Table 2, only 0.6% of diabetic patients without any ACEs demonstrated inadequate self‐care behaviors, while a significant 47.8% of those with five or more ACEs reported a craving for smoking. Conversely, among female students devoid of ACEs, 27.3% exhibited self‐care behaviors that were below the recommended level, suggesting a higher adherence to diabetes self‐care among this demographic. The Chi‐square test (Mantel‐Haenszel trend test) indicated a significant variation in the percentage of diabetic patients exhibiting self‐care behaviors based on their ACEs (*χ* ^2^ = 34.07, *p* < 0.01).

**Table 2:** The association between ACEs and self‐care behaviors in diabetic patients.

Number of ACEs	Percentage (%) of diabetic patients with self‐care behaviors according to their ACEs	
	Above the cut‐off (more diabetes self‐care behavior)	Bellow the cut‐off (less diabetes self‐care behavior)
No ACEs	18.9	0.6
One to four ACEs	42	51.7
Five or more ACEs	39.1	47.8

*Note: χ* ^2^ Mantel‐Haenzsel test for trend = 34.07, *p* < 0.01.

### 3.3 Logistic Regression Analysis

To investigate the association between patient demographics, socioeconomic factors, mindfulness practices, and ACEs with the likelihood of engaging in self‐care behaviors, a multivariate logistic regression analysis was conducted. Table 3 indicated that after accounting for demographic variables, patients with one to four adverse exposures were more inclined to exhibit poor self‐care behaviors compared to those without any adverse exposures (odds ratio [OR]: 0.38; 95% confidence interval [CI]: 0.17–0.63, *p* < 0.005), In other terms, individuals who experienced between one and four ACEs demonstrated a 62% reduction in the likelihood of engaging in self‐care behaviors. For those with six or more ACEs, this reduction increases to 68% (OR: 0.32; 95% CI: 0.15–0.78, *p* < 0.005). Our research indicates a significant correlation between subjective SES and self‐care practices. Individuals reporting a higher subjective SES demonstrated a 35% greater likelihood of engaging in self‐care behaviors (OR: 1.35, 95% CI: 1.16–1.58, *p* < 0.005). Furthermore, mindfulness was also significantly associated with self‐care behavior, increasing the odds of reporting such practices by 12% (OR: 1.12, 95% CI: 1.06–1.19, *p* < 0.005).

**Table 3:** Age, ACEs, subjective socioeconomic status, mindfulness, and adjusted odds ratios of self‐care behaviors in Type 2 diabetic patients.

Variables	Self‐care behaviors	
	Crude OR (95% CI)	Adjusted^a^ OR (95% CI)
Age	1.05 (1.01–1.09)	1.10 (1.05–1.16)
Subjective socioeconomic status	1.20 (1.06–1.36)	1.35 (1.16–1.58)
Mindfulness	1.16 (1.11–1.21)	1.12 (1.06–1.19)
1–4 ACEs	0.45 (0.24–0.83)	0.38 (0.17–0.63)
6 or more ACEs	0.21 (0.10–0.47	0.32 (0.15–0.78)
No ACEs	Reference	

^a^ Adjusted for gender, marital status, education of woman, and education of husband.

## 4 Discussion

This study aimed to investigate the relationship between self‐care behaviors in Type 2 diabetic patients in Isfahan, Iran, and their experiences with adverse childhood events, mindfulness, and subjective SES. The results revealed that over 33% of the study participants had experienced five or more adverse childhood events. Our research aligns with the findings of Alhowamle et al. [43], who reported that 57.9% of participants with chronic illnesses, predominantly Type 2 diabetes, experienced four or more ACEs. This aligns with the growing body of literature indicating that ACEs are prevalent within the general population. Furthermore, these findings suggest that ACEs may be as impactful for individuals with diabetes as depression, a condition often recognized as significantly affecting this demographic. Notably, research has indicated that ACEs are experienced by 14% of youth and 12%–13% of adults [44, 45].

The study revealed that a significant majority (50.9%) of diabetic participants exhibited suboptimal self‐care practices. This finding aligns with previous research conducted by Al‐Qahtani et al. [46], which reported that a substantial proportion (90.1%) of the Saudi Arabian population demonstrates inadequate self‐care behaviors in managing diabetes. This suggests a widespread need for improved patient education and support systems to promote effective self‐management strategies among individuals with diabetes. A systematic review of research conducted in Ethiopia [47] and low‐ to middle‐income countries [48] indicates a concerning trend of suboptimal self‐care practices among individuals with Type 2 diabetes.

The psychosocial model of development highlights the profound impact of childhood experiences on adult well‐being. Specifically, child abuse during formative years can have detrimental effects on the nervous, immune, and endocrine systems [49]. Chronic stress, often associated with ACEs, can have profound implications for physical and mental health. When biological systems develop under persistent stress, the functioning of nervous, immune, hormonal, and other systems is disrupted. This disruption can contribute to the development of various physical and mental illnesses. Furthermore, prolonged exposure to toxic stress has been linked to substance abuse, eating disorders, and depression, all of which are known to impede effective diabetes self‐management [50]. Research indicates that individuals exposed to four or more ACEs demonstrated a significantly higher risk of developing chronic diseases, with an increase ranging from 2 to 11 times. Additionally, the likelihood of engaging in risky health behaviors related to diabetes was found to be elevated by a factor of 8–21 [51].

ACEs are believed to induce enduring biological and psychological alterations that contribute to adverse health consequences [52, 53], thereby offering a plausible rationale for the notable correlation between ACEs and chronic conditions, including Type 2 diabetes.

Regarding Mindfulness, our research indicated that mindfulness serves as a protective element influencing the likelihood of self‐care behaviors among individuals with Type 2 diabetes, with patients exhibiting a 12% increased propensity to participate in self‐care activities. This outcome aligns with findings from previous studies conducted in Iran [54, 55]. Drawing upon the principles of cognitive‐behavioral therapy, intervention strategies aimed at modifying and confronting irrational beliefs, coupled with enhanced patient attention, can significantly improve adherence to diabetes self‐care programs. This approach fosters increased self‐awareness and encourages patients to actively engage in treatment protocols [56]. MBIs have demonstrated a positive impact on numerous aspects of diabetes management, encompassing biological, psychological, and social domains [57]. In patients with diabetes, non‐compliance with treatment, irregular monitoring of blood sugar, and incorrect treatment lead to an increase in blood sugar and increased patient anxiety. As other studies have shown, practicing mindfulness can help to improve blood sugar control [58]. Besides, Mindfulness practices have demonstrated the potential to enhance self‐care habits, bolster self‐confidence, and foster improved self‐regulation. These positive effects can contribute to a more fulfilling and enriching quality of life [59].

Our research indicates a positive correlation between diabetic patients' subjective SES and their engagement in self‐care behaviors. This finding aligns with previous studies, suggesting a strong link between an individual's perception of their socioeconomic standing and their proactive health management practices [60]. The social and cultural attributes associated with various social classes appear to contribute to variations in social identity formation and self‐conception, thereby impacting the choices and behaviors of individuals [61, 62].

The results of our research indicate that a higher subjective SES among diabetic patients is positively correlated with improved self‐care behaviors. This outcome is consistent with prior studies [63]. In individuals with diabetes, subjective SES is linked to behaviors such as engaging in physical activity and consuming fruits and vegetables [64]. Additionally, it plays a role in glycemic control [65] and, as a result, is connected to an individual's self‐care practices. In alignment with our research findings, Berkowitz et al. [66], found that individuals reporting a higher subjective SES were more inclined to adhere to a nutritious diet, consequently resulting in improved HbA1c levels. Conversely, individuals with lower SES often encounter a series of detrimental living conditions that contribute to elevated chronic stress levels [67].

## 5 Limitations

The present study is not without its limitations. It is important to recognize that when evaluating subjective SES, the findings may be affected by the individual's self‐assessment of their financial circumstances, potentially overlooking additional variables such as objective measures or family background. Furthermore, the study is subject to recall biases, restrictions on broader generalization, and an inability to examine the temporal relationships between outcome and risk factors, all of which are inherent limitations of the cross‐sectional design employed in this study.

## 6 Conclusion

This study suggests that psychosocial factors like mindfulness and perceived SES may positively influence self‐care behaviors in individuals with Type 2 diabetes. The findings indicate that incorporating MBIs into diabetes treatment plans could be a beneficial strategy to enhance self‐care adherence. These results highlight the potential for using mindfulness exercises to empower individuals with diabetes to actively manage their health. Our research indicates a correlation between ACEs and decreased self‐care practices among individuals with Type 2 diabetes. This finding suggests significant policy implications, highlighting the necessity for a collaborative approach involving multiple agencies in healthcare and social services. Such an effort is essential for screening, preventing, and delivering early intervention for those affected by ACE. Given that adherence to self‐care practices can help prevent the morbidities and mortalities associated with Type 2 Diabetes, a key implication for practitioners is to design and implement educational and awareness initiatives aimed at enhancing self‐care behaviors. Future research should focus on examining the influence of objective socio‐economic factors, as well as exploring additional psychosocial elements that may affect self‐care practices in individuals with diabetes. Furthermore, it is recommended to study the effectiveness of cultural and community‐based interventions in enhancing self‐care behaviors. Further investigation into the efficacy of mindfulness meditation through rigorous, well‐designed studies is warranted to fully understand its potential benefits.

## Author Contributions

Mehdi Nosratabadi designed the study, drafted the manuscript, and approved the final manuscript.

## Ethics Statement

The study received the required ethics approval from the Isfahan University of Medical Sciences Research Ethics Committee, Isfahan, Iran, with Ethics Code No. IR.MUI.REC.1399.373.

## Consent

Participants sign a written informed consent in which they have been assured that their identities and responses will be kept anonymous and that participants' data will be kept as confidential as possible.

## Conflicts of Interest

The author declares no conflicts of interest.

## Transparency Statement

The lead author, Mehdi Nosratabadi, affirms that this manuscript is an honest, accurate, and transparent account of the study being reported; that no important aspects of the study have been omitted; and that any discrepancies from the study as planned (and, if relevant, registered) have been explained.

## Data Availability

The datasets used and/or analyzed in this study are available from the corresponding author upon reasonable request.
